# Supplementary material for: StrainCascade: An automated, modular workflow for high-throughput long-read bacterial genome reconstruction and characterization
Source: iScience. 2026 Jun 4;29(6):116189. doi: 10.1016/j.isci.2026.116189 (PMC13266145; doi:10.1016/j.isci.2026.116189)
Supplement: Document S1. Figures S1–S6 [file mmc1.pdf]

iScience, Volume *29*

Supplemental information

StrainCascade: An automated, modular workflow for high-throughput long-read bacterial genome reconstruction and characterization

Sebastian B.U. Jordi, Isabel Baertschi, Jiaqi Li, Nadia Fasel, Benjamin Misselwitz, and Bahtiyar Yilmaz

SUPPLEMENTAL FIGURES

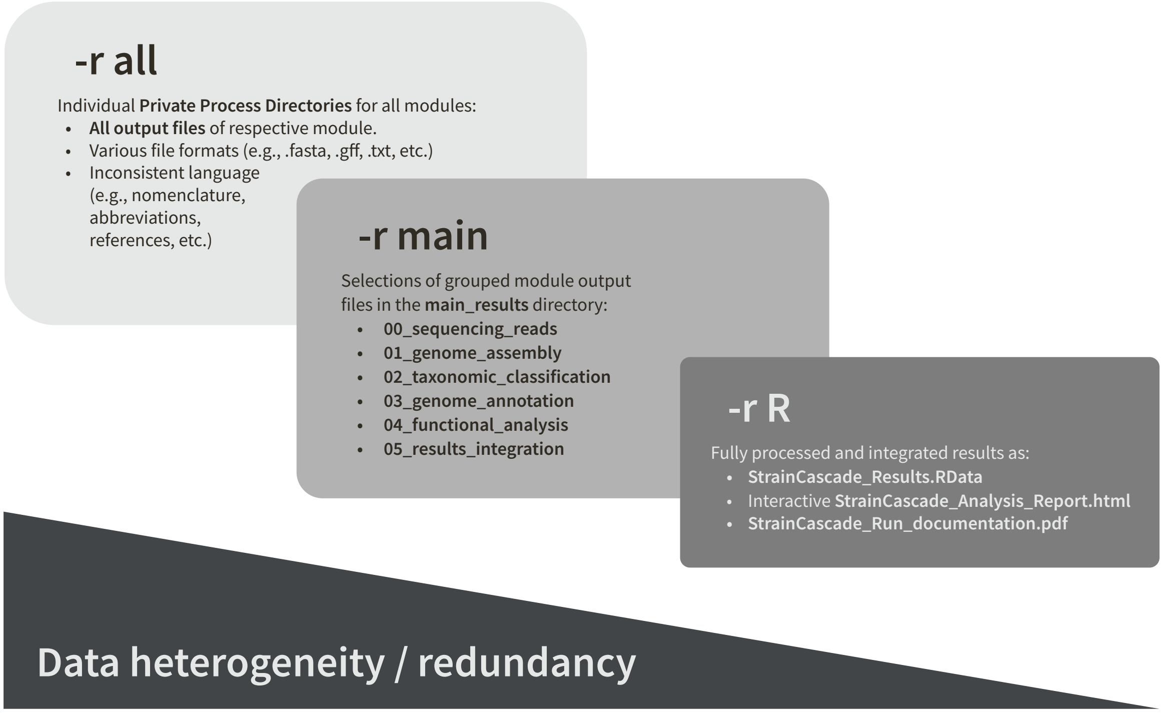

Figure S1. The execution framework of *StrainCascade* ensures reproducibility and structured data management. Each module operates within an isolated Private Process Directory (PPD), systematically managing intermediate and final files to maintain workflow integrity. Users can customize result retention granularity with three options: *all*, *main*, or *R*. The pipeline has the option of deterministic execution with controlled entropy sources, ensuring reproducibility across computational environments. Automated cryptographic integrity verification (SHA-256 hashing) safeguards data consistency throughout the workflow. Additionally, *StrainCascade* supports adaptive multi-threading execution, optimizing resource allocation based on system availability. This structured computational framework enables seamless scalability from single-genome assembly to large-scale comparative genomics while maintaining full documentation of pipeline execution for enhanced transparency.

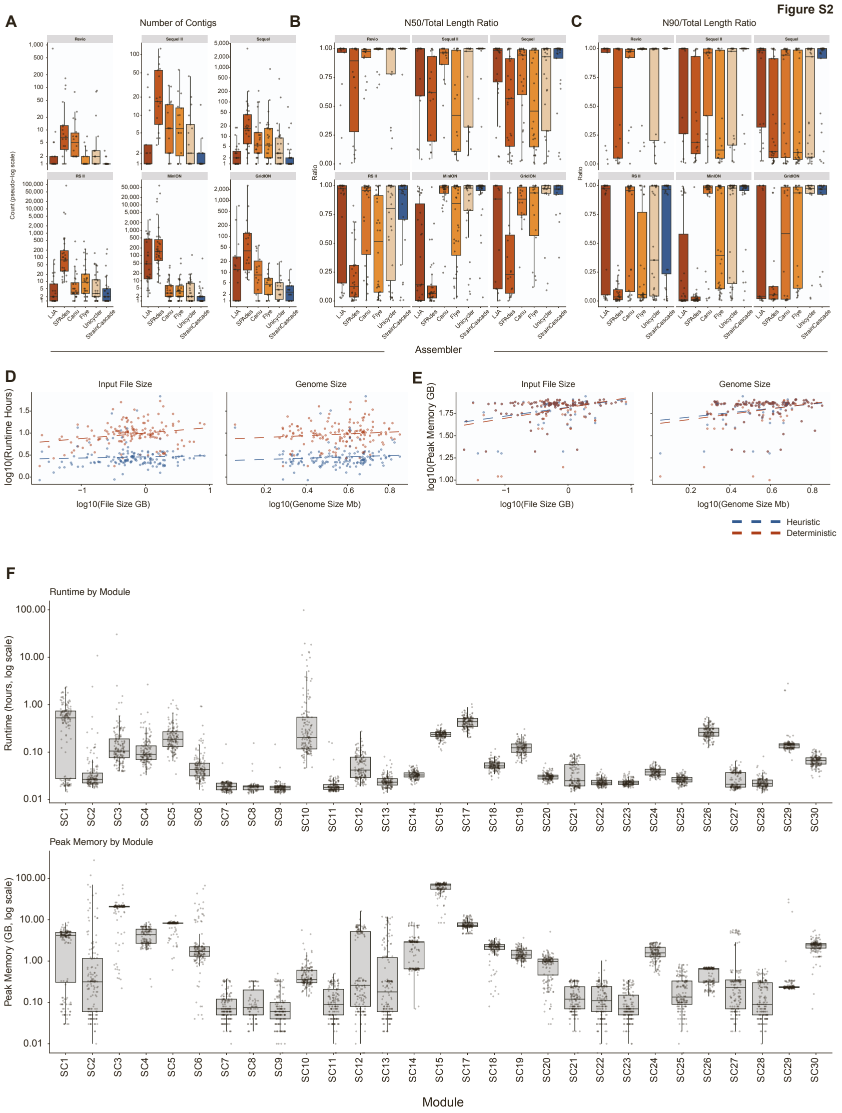

Figure S2. Benchmarking *StrainCascade* across assemblers, sequencing platforms, and computational performance parameters.

(A) Boxplots showing the number of contigs for assemblies generated by La Jolla Assembler (LJA), SPAdes, Canu, Flye, Unicycler, and StrainCascade across PacBio Revio, Sequel II, Sequel, and RS II, and Oxford Nanopore MinION and GridION datasets.

(B) Boxplots showing the N50-to-total-length ratio for assemblies generated by La Jolla Assembler (LJA), SPAdes, Canu, Flye, Unicycler, and StrainCascade across PacBio Revio, Sequel II, Sequel, and RS II, and Oxford Nanopore MinION and GridION datasets.

(C) Boxplots showing the N90-to-total-length ratio for assemblies generated by La Jolla Assembler (LJA), SPAdes, Canu, Flye, Unicycler, and StrainCascade across PacBio Revio, Sequel II, Sequel, and RS II, and Oxford Nanopore MinION and GridION datasets.

(D) Relationship between input file size (log_{10} scale) and *StrainCascade* runtime (log_{10} hours), and relationship between genome size (log_{10} Mb scale) and *StrainCascade* runtime (log_{10} hours).

(E) Relationship between input file size (log_{10} scale) and peak memory usage (log_{10} GB) with *StrainCascade*, and relationship between genome size (log_{10} Mb scale) and peak memory usage (log_{10} GB) with *StrainCascade*.

(F) Boxplots showing runtime (top) and peak memory usage (bottom) for individual *StrainCascade* modules (SC1-SC30) across analyzed datasets. Values are displayed on a log scale to capture the broad range of computational demands across modules
The error bars represent standard deviation.

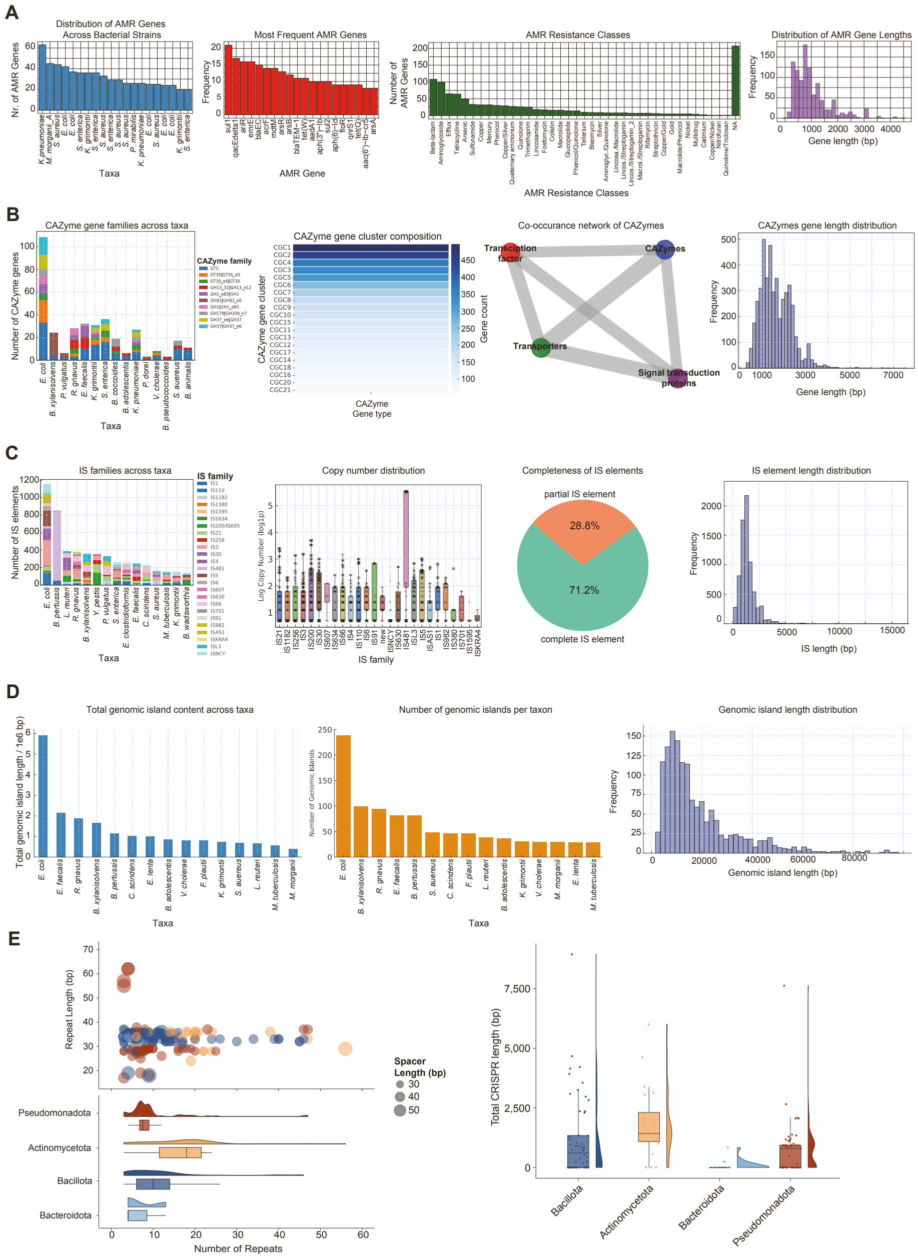

Figure S3. Detection of genomic functional features across bacterial strains.

(A) AMR genes are detected across bacterial strains, revealing strain-specific variations in abundance, identifying the most prevalent resistance genes, classifying them into resistance classes, and analyzing their length distribution.

(B) CAZyme genes are analyzed across bacterial taxa, revealing their distribution, co-occurrence with transporters, transcription factors, and signaling proteins, gene length diversity, and the composition of CAZyme gene clusters, highlighting key functional associations.

(C) Insertion sequences (IS) are analyzed across bacterial taxa, examining their distribution, copy number variations, proportion of complete versus partial elements, and length diversity across strains.

(D) Genomic islands are analyzed across bacterial taxa, assessing their total content per taxon, species-specific abundance, and length distribution, providing insights into genomic plasticity and adaptation.

(E) CRISPR spacer distribution and cross-genome conservation are shown using the number of repeats and repeat length (bp) across major bacterial phyla, including Pseudomonadota, Bacteroidota, Actinomycetota, and Bacillota (left panels). Box-dot plot with a histogram showing the total CRISPR length (bp) distribution across these major bacterial phyla (right panel).

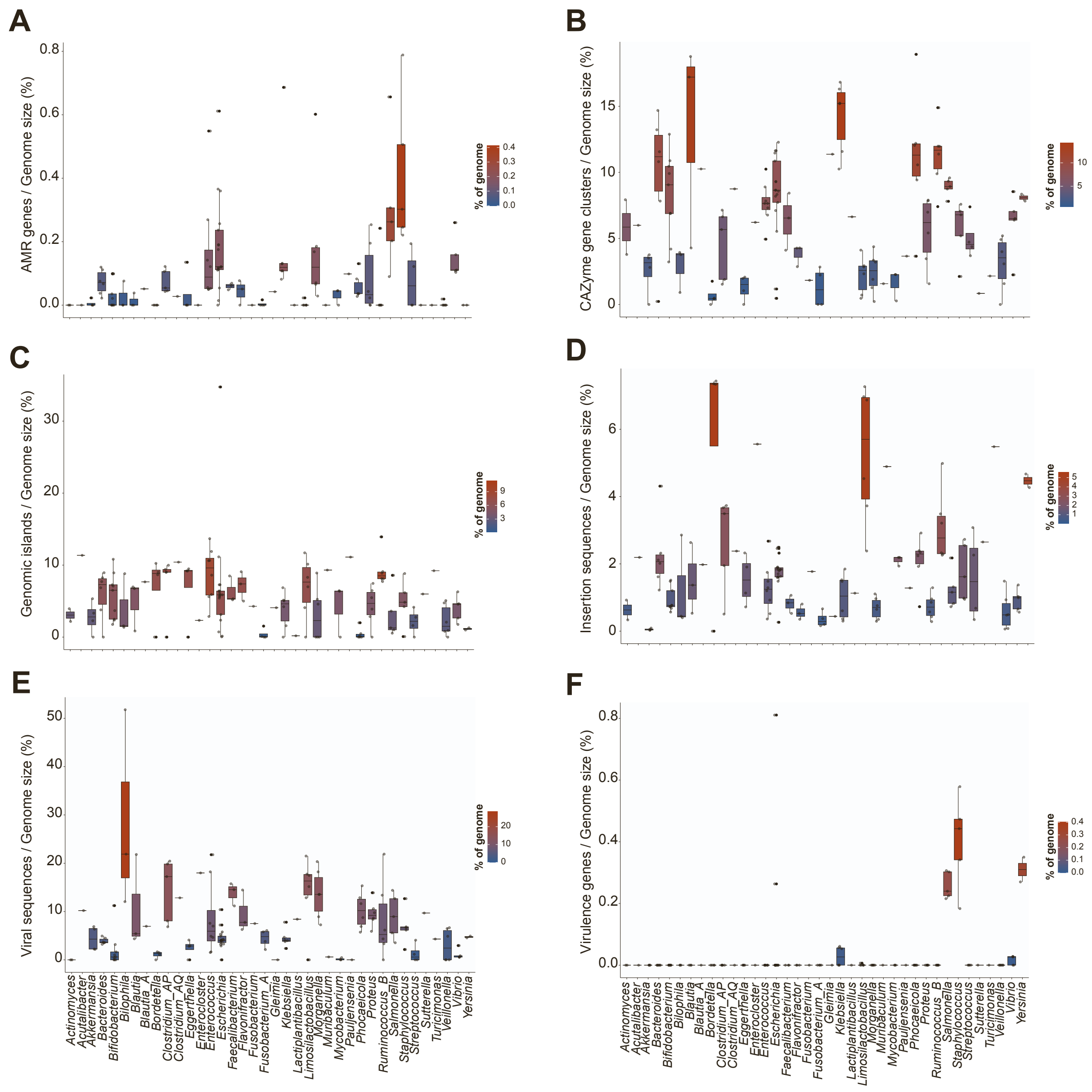

Figure S4. Taxa-specific variability in functional and mobile element distribution.

(A) Proportion of AMR genes per genome size across bacterial taxa, highlighting variability in resistance potential among different species.

(B) CAZyme gene cluster density, showing the relative abundance of carbohydrate-active enzyme clusters across genomes.

(C) Genomic islands per genome size, illustrating the distribution of horizontally acquired gene regions across different taxa.

(D) Insertion sequence elements per genome size, reflecting the extent of transposable elements contributing to genome plasticity.

(E) Viral sequences per genome size, showing variation in the prevalence of viral insertions among bacterial genomes.

(F) Virulence gene density, indicating differences in the genetic potential for pathogenicity across species.

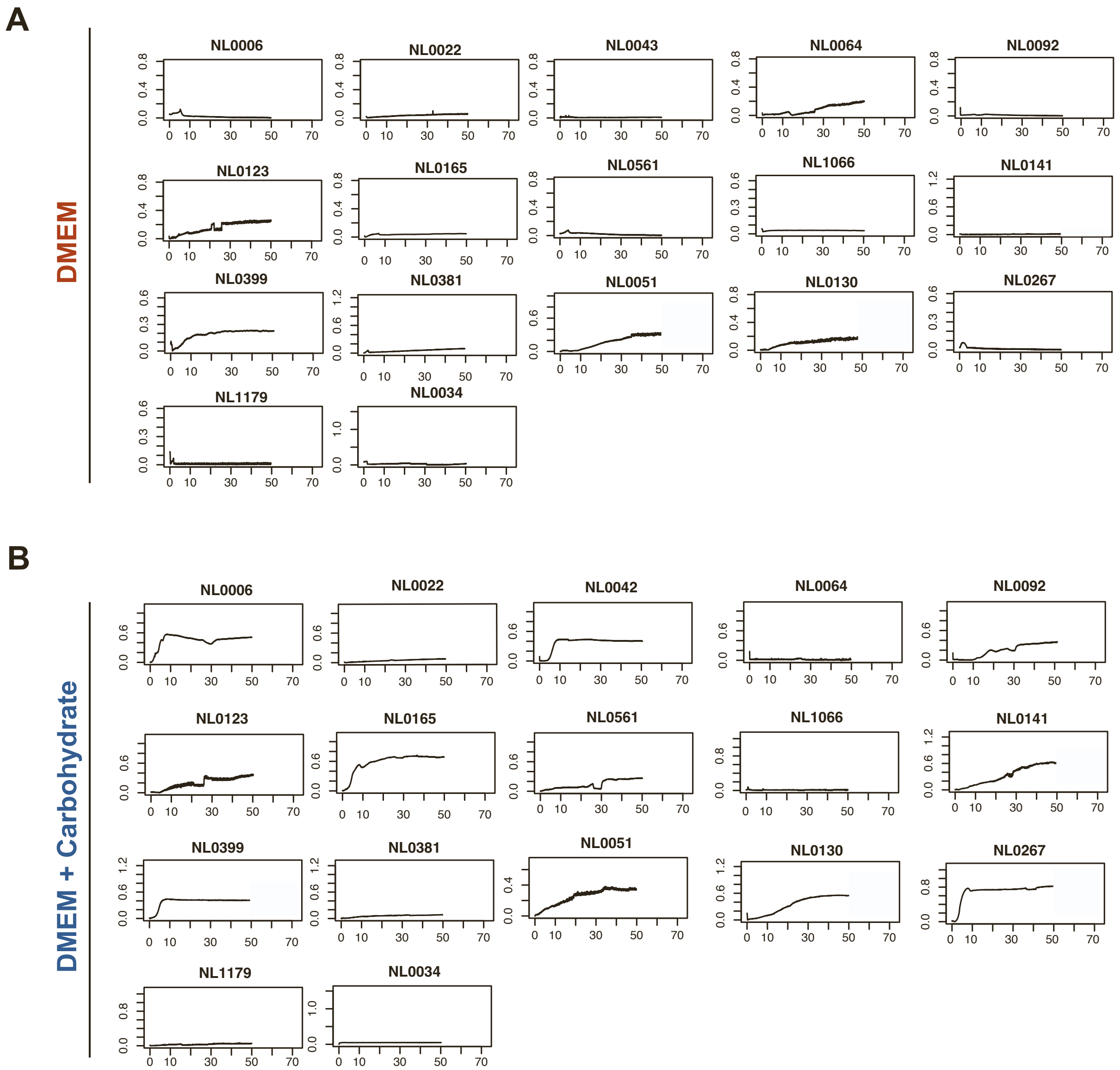

Figure S5. Growth dynamics of in-house bacterial isolates with and without carbohydrate-supplemented media.

(A) Growth curves of bacterial isolates cultured in DMEM alone.

(B) Growth curves of the same isolates cultured in DMEM supplemented with carbohydrates. Optical density (OD600) was monitored over time (hours) to assess growth dynamics and metabolic adaptability. Each panel represents an individual isolate. Strains harboring a higher proportion of carbohydrate-active enzyme (CAZyme) genes generally displayed enhanced growth in carbohydrate-supplemented medium. Growth curves represent single measurements per strain and condition and are shown for descriptive comparison only.

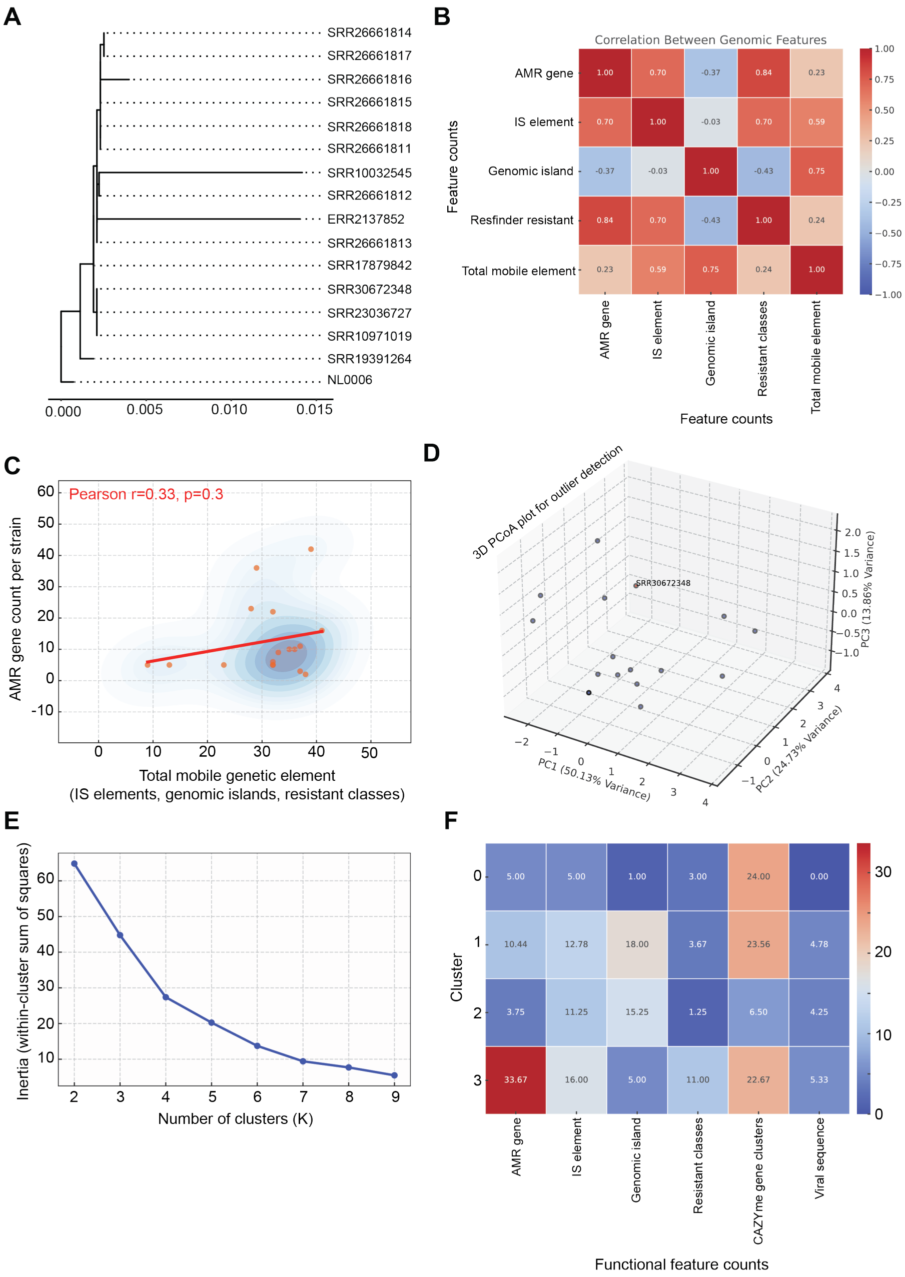

Figure S6. Functional and evolutionary classification of *E. coli* strains.

(A) Phylogenetic resolution of *E. coli* strains used in this study with their corresponding Sequence Read Archive (SRA) number.

(B) The heatmap illustrates the correlation between key genomic features across *E. coli* strains, highlighting relationships between AMR genes, mobile genetic elements, and genomic islands.

(C) Scatter plot shows the relationship between AMR gene load and mobile genetic elements (insertion sequences, genomic islands, and resistant classes). Density contours illustrate the distribution of strains, showing no correlation.

(D) A 3D Principal Coordinates Analysis (PCoA) plot identifies functionally distinct outliers. For one strain significantly deviating from the main clusters, the Sequence Read Archive (SRA) number is plotted, indicating potential adaptive or horizontally transferred genomic elements and highlighting genomic plasticity.

(E) The Elbow method plot shows the within-cluster sum of squares (inertia) vs. the number of clusters (K). The inflection point (elbow) at K=4 suggests an optimal cluster number, balancing variance reduction with model complexity.

(F) The heatmap shows the functional classification of *E. coli* strains using unsupervised clustering based on key genomic and metabolic features. Strains were grouped into four distinct functional clusters (0-3), highlighting differences in AMR, mobile genetic elements, and metabolic potential, including CAZymes.
